# Necrotising Fasciitis: Appearances Can Be Deceptive

**DOI:** 10.29252/wjps.10.1.43

**Published:** 2021-01

**Authors:** Shanthakumar Shivalingappa, KN Manjunath, Veena Waiker, M Kumaraswamy, Udayashankar Odeyar

**Affiliations:** 1 Department of Plastic and Reconstructive Surgery, Ramaiah Medical College, Bangalore, India;; 2 Plastic Surgeon, Bangalore, India

**Keywords:** Necrotising fasciitis, Streptococcus, Carcinoma, Reconstruction

## Abstract

**BACKGROUND:**

Necrotizing fasciitis is a potentially fatal infection of β hemolytic Group-A Streptococcus, often occurring in patients with other comorbidities, but can occur in healthy individuals as well. It commonly affects the extremities, perineum, and abdominal wall. The aim of this study was to highlight various presentations of necrotizing fasciitis in unusual anatomical sites with delayed diagnosis and treatment.

**METHODS:**

In a retrospective analysis, seven cases of unusual presentations of necrotizing fasciitis were enrolled during a period of five years treated in a tertiary centre.

**RESULTS:**

The patients were between 23 and 80 years. Four were males and three were females. Four out of seven were diabetic. All patients had septicemia (hypovolemic shock, with leucocytosis, thrombocytopenia and deranged coagulation parameters) on admission in the intensive care unit. All seven patients had minimal cutaneous manifestation and the remote primary pathology was diagnosed in two patients. Six patients out of seven survived and the morbid state continued in one patient in view of malignancy of rectum in one patient. The overall outcome was satisfactory in five out of seven cases.

**CONCLUSION:**

Pain disproportionate to the local inflammation with florid constitutional symptoms should raise suspicion of necrotizing fasciitis. Early diagnosis, of stabilization of hemodynamics, emergency fasciotomy, staged debridement and the initiation of broad spectrum antibiotics reduced the morbidity and mortality. The disease may manifest with uncommon presentations and sometimes lead to the diagnosis of primary aetiology.

## INTRODUCTION

The discovery of necrotising fasciitis dates back to Hippocrates in 5^th^century B.C. and Wilson in 1952.^1^ The early clinical diagnostic challenge still lurks despite adjunct radiological and laboratory investigations; hence, the morbidity and mortality are high, even today. Necrotizing fasciitis is a rapidly progressive spreading inflammation caused by Group-A β hemolytic Streptococcus, but may be polymicrobial.^2^^,^^3^ The major risk factors are diabetes, peripheral vascular disease, old age and immune-compromised status but can primarily affect healthy individual too.^4^

Surgical procedures, post-cesarean section and minor wounds are usual pre-disposing factors. The extremities, abdominal wall and perineum are more commonly affected than other regions.^5^ The organism gains entry through a breech in the skin and initiates local infection seen as skin blisters. Infection rapidly progresses along fascial planes involving large surface areas and depth. Muscle compartments are affected as a consequence of spreading infection, but the cutaneous manifestations are limited. Constitutional symptoms like severe pain out of proportion to the local inflammation in the affected area, high grade fever, vomiting and diarrhea should raise suspicion of necrotizing fasciitis. Successful outcome of treatment depends on differentiating limb threatening infection like necrotizing fasciitis, from non-limb-threatening superficial cellulitis.^6^

Though the incidence of necrotizing fasciitis is high, a retrospective observation of few patients with unusual presentations and involvement of uncommon anatomical regions has enabled us to view the disease with a much wider perspective which would otherwise be missed. The aim of presenting this study was to highlight the uncommon acute presentations of necrotizing fasciitis at uncommon sites, which may sometimes downplay the severity and mislead the diagnosis, thereby delaying the time of surgical intervention resulting in increased morbidity and mortality. Also to emphasize on a thorough clinical examination supported by laboratory and imaging studies in patients with uncommon presentations, which would throw light on unusual etiological factors that enables the surgeon to customize the management.

## MATERIALS AND METHODS

All patients admitted from 2009 to 2014 were included in the study. The patients with the diagnosis of necrotizing fasciitis in unusual sites were enrolled. On admission, thorough history was taken and details of previous hospitalisation were obtained. The local manifestations were recorded. The findings during the surgery were noticed. Patient’s sepsis parameters were prepared. The severity of infection in comparison to the amount of local tissue damage was studied. Management was done with intravenous clindamycin, 600 mg twice a day for 48 hours, and then continued with 300 mg of the medication 12^th^ hourly for one week to ten days in all patients in addition to other antibiotics according to the sensitivity report.

All procedures performed in the study involving human participants were in accordance with the ethical standards and with the 1964 Helsinki declaration and its later amendments or comparable ethical standards. For retrospective studies, a formal consent was not required and provided. Surgical management included multiple surgical debridements. The features suggestive of necrotising fasciitis like necrotic fascia and easy separation of the fascia from the overlying skin were looked for. On easy separation of the skin from the fascia, the diagnosis of necrotising fasciitis was confirmed. Factors affecting the recovery of the patients like duration of hospital stay, time taken for sepsis parameters to improve, and the number of undertaken reconstructive procedures were all recorded. The patient was deemed recovered, when the patient was fit for discharge. The continuous variables were described as mean and median with standard deviations.

## RESULTS

The age of the affected patients was not limited to one group. The youngest patient was 23 years old and the oldest was 83 years old. Before coming to our hospital, 7 patients (100%) had history of treatment outside the hospital. Patients were referred to our hospital after a median time of 5.8 days from the time of onset of symptoms. Four out of 7 patients (57.1%) were diabetics and had sustained trivial injuries. All patients were diagnosed as cellulitis and took conservative treatment in other hospital (Table 1). All seven patients (100%) had septicemia on admission with deranged laboratory parameters (Table 2).

On exploration, non-viable deep tissue and fascia were found through the wounds in all patients (100%); hence, the diagnosis of necrotizing fasciitis was confirmed. 

On debridement, all patients had necrosis of superficial and deep fascia, epimysium and fascial coverings of neurovascular structures that could be easily dissected and excised (Table 3). Group a Beta hemolytic Streptococcus was isolated in two patients, polymicrobial in three, one had methicillin sensitive *Staphylococcus aureus* and there was no growth in one patient. 

**Table 1:** Primary diagnosis at peripheral hospital and management

**Name**	**Age (Years)**	**Sex**	**Reported to hospital**	**Primary diagnosis in peripheral hospital**	**Primary treatment** **in peripheral hospital**	**Co-morbidities**
C1 (Figure 1)	23	F	After 2 days fever, pain in left breast	Breast abscess	I & D	Local factors
C2 (Figure 2)	50	M	After 2 days Fever, pain in neck	Laceration Left side chin and cellulitis	Antibiotics	Diabetes mellitus
C3	55	F	After 4 days fever, pain, swelling in perineum	Perianal abscess, cellulitis	I &D, Antibiotics	Malignancy
C4	80	M	After 2 days pain in left eye, fever	Cellulitis following laceration on the upper eye lid	Antibiotics	Diabetes mellitus, hypertension, left hemiparesis
C5	40	M	After 4 days pain in the back and left foot, fever	Gangrene, cellulitis of left great toe and back	I &D, Antibiotics	Diabetes mellitus and anasarca
C6 (Figure 3)	52	F	After 4 days pain, facial edema, fever	Scalp ulcer with cellulitis of face	Antibiotics, conservative management for facial cellulitis	Diabetes mellitus
C7	55	M	Patient did not visit any hospital for 10 days.	-	Treated at home with analgesics and anti-pyretic.	Nil

**Fig. 1:**
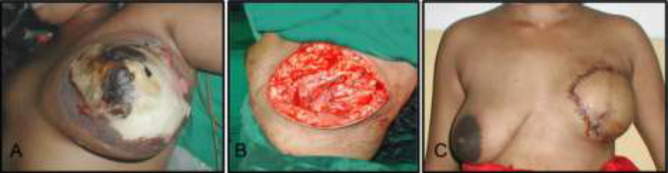
A 23 years old patient referred to the hospital After 2 days of fever and pain in left breast with primary diagnosis of breast abscess undergoing I&D treatment

**Fig. 2:**
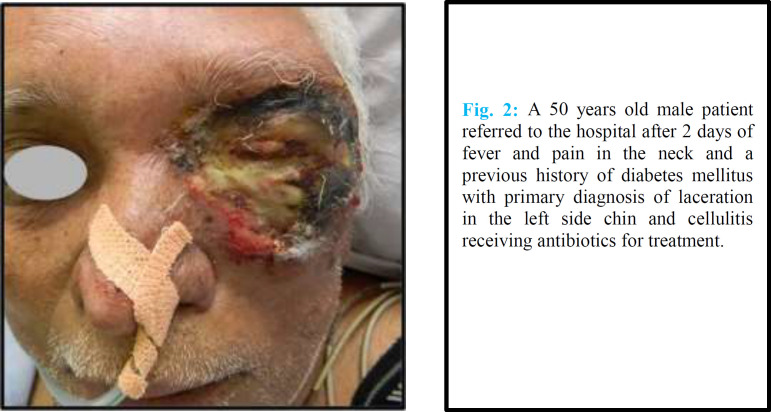
A 50 years old male patient referred to the hospital after 2 days of fever and pain in the neck and a previous history of diabetes mellitus with primary diagnosis of laceration in the left side chin and cellulitis receiving antibiotics for treatment

**Fig. 3:**
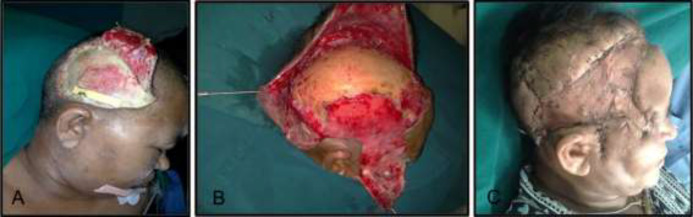
A 52 years old female patient referred to the hospital after 4 days of fever, pain and facial edema and a previous history of diabetes mellitus with primary diagnosis of scalp ulcer and face cellulitis receiving antibiotics and conservative management for facial cellulitis

**Table 2:** Lab parameters on admission to our hospital

**Name**	**Hb** **g/dL**	**TC ** **Cells/cu.mm**	**Platelet**	**APTT** **secs**	**Creat**	**Alb** **G/dL**	**Culture**	**Radiology**
C1	8	29,990	40,000	40.1	2.0	1.8	Group A Beta haemolytic. Streptococcus	
C2	13.1 pcv-41	20,000	10,000	53.1	1.8	2.5	Group A Beta haemolytic Streptococcus	MRI Inflammation neck and chest. Thrombosis of external jugular vein
C3	5.4	17,700	80,000	39		1.4	*K. oxytoca* *E. Coli* Gram positive cocci	
C4	7.0	19,000	1,00,000	36.4	1.9	2.0	No growth	C.T. scan Soft tissue swelling of periorbital, prefrontal, premaxillary, perizygomaticareas. C.T. scan brain, old subcortical lacunar infarct. M.R.I. of cavernous sinus region, normal.
C5	8	12,000	60,000	41.3	2.0	2.0	K. pneumonia, Acinitobacter, *P. aeruginosa*, Enterobacter, Saureus (MSSA)	C.T. scan abdomen, chronic pancreatitis
C6	9.7	23,420	1.93 lakhs	39.5	1.07	1.22	*K. pneumonia* Acinitobacter	
C7	1.4	44.8	1.57		1.6		MSSA	Bilateral minimal pleural effusion, fluid in left ischiorectal fossa, diffuse subcutaneous oedema in left gluteal region, dilated proximal small bowel loops with collapse of distal ileal loops suggestive of acute intestinal obstruction.

**Table 3:** Late clinical presentation at our hospital

**Name**	**Day of** **presentation**	**Extent**	**HR/min**	**BP mm/Hg**	**Temp** ^0^ **F**
C1	After 1 week	Left Breast	110	90/50	102
C2	After 5 days	Right side of neck and chin	100	100/74	100
C3	After 1 week	Lower abdomen and right thigh	114	90/60	101
C4	After 2 days	Right eyelids and peri-orbital area	100	100/50	110
C5	After 1 week	Back of trunk, left leg and great toe	106	100/60	101
C6	After 1 week	Right fronto-temporo-parieto-occipital areas and face	110	100/80	101
C7	After 10 days	Left ischial area, scrotum, both inguinal regions and lower abdominal wall up to umbilicus	110	116/70	98.6

This was followed by wound bed preparation and resurfacing after mean time of 9.8 days. Multiple surgeries for optimization of wound bed were shown in Table 4. Finally, out of 7 patients, six survived and one was expired due to MODS. Out of the seven patients, six survived and the patient with associated chronic pancreatitis was expired due to septicemia and acute respiratory distress syndrome (Table 4).

**Table 4:** Management at our hospital

**Name**	**Surgical procedure**	**Day of wound cover**	**Outcome**	**Uncommon presentation**	**Uncommon site**
C1	Left mastectomy and reconstruction with RT pedicled TRAM flap	After 2 days	Recovered well.	Lactating woman with Cracks & fissures of nipple on breast pump	Breast
C2	Staged debridements and SSG	After 7 days	Hoarseness of voice recovered by 3 months	Superficial 2 cm laceration on chin while shaving at barber shop	Neck and chin
C3	Permanent colostomy , multiple debridements+SSG	After10 days	Treated conservatively with permanent colostomy	Presented as perianal abscess, PR-Growth in rectum, HPE, Adenocarcinoma of rectum	-
C4	Multiple conservative debridement of upper and lower eyelids and periorbital area+SSG	After 10 days	Recovered	Superficial laceration on eyelid leading to full thickness necrosis of orbital area	Eyelids and peri-orbital area
C5	Multiple debridement, disarticulation of left great toe, tibialis anterior muscle flap to cover middle 3^rd^ tibia+ SSG on back and left leg	After 21 days	Expired	Associated Chronic pancreatitis, hypoalbuminemia, anasarca Extensive, pale, edematous wounds which never healed. Flap and SSG failed.	Back of trunk
C6	Multiple debridement, scalp advancement flap and SSG	After 9 days	Scar contracture lateral canthus of right eye	Undetected painless post traumatic necrotic patch in right temporoperietal region of scalp with extensive necrosis of pericranium, parotid fascia and SMAS	Scalp and SMAS
C7	Multiple debridement+SSG	Extensive wound debridement and split skin graft.	Recovered well.	Presented with features of acute abdomen	**-**
					

## DISCUSSION

Necrotising fasciitis though rare, is known for its high morbidity and mortality. Its similarity to other cutaneous inflammation poses a huge diagnostic challenge even today. However, early diagnosis is the key for better outcome as early initiation of appropriate antibiotic and surgical therapy. Etiological factors are often trivial like trauma, cutaneous wounds, drug injections, abscesses, tinea-pedis in the lower extremity, and animal or insect bites.^7^ Hematogenous spread of microorganisms from a distant site of infection can sometimes be detected even with small injuries.^8^

The organisms usually enter the body through a minor wound like our patients (C1, C2 and C4) who had small wounds on the breast, chin and eyelid, respectively; which is consistent with other studies.^9^ The disease spreads rapidly along tangential planes through fascia with extensive undermining with apparently normal looking skin. The condition evolves, through the following clinical stages which is helpful in assessment of severity and progress of the disease. Stage 1: Pain, tenderness and warm skin; Stage 2: Blister or bulla formation which signals the onset of critical skin ischemia; and Stage 3: Onset of tissue necrosis characterized by the ‘hard signs’ of necrotizing soft tissue infection, such as hemorrhagic bullae, skin anesthesia and frank skin gangrene.^7^

The classical systemic manifestations like high fever, severe excruciating pain in the affected region and prostration should raise suspicion of necrotizing fasciitis in the early stages as diagnosis is often missed in this stage.^10^ All patients were diagnosed to have cellulitis when they first attended the referral hospital. Even in other studies, the majority were diagnosed as cellulitis with a differential diagnosis of erysipelas, myonecrosis, lymphedema, eosinophilic fasciitis, phlegmasia cerulean dolens, or myxedema.^11^^-^^13^ Hence, the management in the initial stages was less aggressive in our patients which increased the morbidity. 

As described in literature, hypovolemic shock, tachycardia, acidosis, anemia, thrombocytopenia, dyselectrolytemia, hypoalbuminemia, acute renal failure, adult respiratory distress syndrome, altered liver function, deranged coagulation, disseminated intravascular coagulation; multi- organ failure and death are consequences of bacterial systemic toxicity.^14^ All patients were in sepsis by the time they presented to us. The severity of disease severity was dependent on the virulence of the organism and has been classified as Type I: Polymicrobial, where a mixed aerobic and anaerobic infection is the cause (which could be due to *Acetinobacter baumanii* and rarely Vibrio spp. In this situation, blood cultures are positive ~20% of the time.^14^ Three of patients (C3, C5 and C6) had polymicrobial infection.

Type II: Monomicrobial infection is due to Group A Streptococcus and occasionally methicillin-resistant *Staphylococcus aureus* (MRSA). Type II NF is complicated by toxic shock syndrome in up to 50% of cases with positive blood cultures in ~60%.^15^ Two of our patients (C1 and C2) were affected by Streptococcus β hemolyticus who were in toxic shock. The patient C4 was also toxic, but the organism could not be isolated as repeated wound cultures showed no growth. Type III: Gas gangrene infections are most commonly caused by the anaerobe *Clostridium perfringens*.^15^

Some of the scoring systems like the retrospective risk score called LRINEC (Laboratory Risk Indicator for Necrotizing Fasciitis) was calculated by summating values of CRP, total count, hemoglobin, sodium, creatinine and glucose that are complimentary to diagnosis. However, this is not a substitute for clinical judgement.^16^ Laboratory and culture tests have correlative clinical significance.^7^ Imaging studies like computed tomography (CT) is more sensitive and specific in identifying gas and magnetic resonance imaging (MRI) is more sensitive than CT for detecting edema fluid and thickening of fascia. However, it is difficult to differentiate between cellulitis and necrotizing fasciitis with imaging. The patient C2 underwent MRI to assess the depth of the infection in the mediastinal area and in patient C4, CT scan of brain was done to examine the cavernous sinus area.^17^^-^^23^

We also found that these investigations were complimentary to clinical evaluation and were not specific for necrotising fasciitis. Finger test is a simple, bedside diagnostic test as described by Andreasen *et al.* A2 cm incision is made down to the deep fascia under local anesthesia in the area of tenderness or blister. If probing at the level of superficial fascia is undertaken, it reveals lack of bleeding, foul smelling dishwater pus and minimal tissue resistance to finger dissection and it is diagnostic of necrotizing fasciitis; which mandates further debridement.^7^^,^^24^

The primary site of pathology is in the superficial fascia that later is spread to the dermis and deep fascia due to thrombosis and suppuration of the veins and arteries coursing through the fascia.^25 ^All patients had minimal necrosis of overlying skin, but showed thrombosis of vessels and suppuration of fascia and epimysium. Proximal extension of infection along fascia covering neurovascular structures was evident on surgical exploration. The control of infection was indicated clinically by a gradual reduction of constitutional symptoms, bacterial colony count, and improvement of serum albumin and electrolytes. Reduction in wound discharge and edema with growth of vascularized granulation tissue were indicators of progress of the wound bed towards optimisation. All patients were treated with intravenous clindamycin in combination with penicillin group/third generation cephalosporin in addition to surgical excision; which was very effective in reducing the toxic effects within 48 hours except in patients C5 and C6.^24^^,^^26^

Necrotizing fasciitis is more frequent in abdominal wall, perineum and extremities. Involvement of the head and neck structures and especially the scalp is rare in view of good vascularity. In our patients, uncommon areas like breast, chest and neck, back of trunk, orbital area, scalp and abdominal wall were affected and their unusual presentations also misled the diagnosis. Necrotising fasciitis of lactating breast is rare and only a few cases have been reported. It mimics breast abscess and sometimes even carcinoma of breast. Diagnosis may be delayed as the breast and fatty tissue may obscure the inflammation of the pectoral fascia. 

As our patient (C1) was mis-diagnosed to have breast abscess, only small incisions were made for drainage before reporting to our hospital after one week. The delay in wound excision led to widespread infection and ultimately major tissue loss. Literature search revealed the use of split skin graft for resurfacing the post-mastectomy defect; whereas in our case, the breast was reconstructed with contralateral transverse rectus abdominis myocutaneous flap after controlling the infection. It gave psychologically satisfying results to our young patient who was a victim of this benign yet devastating condition.^27^^-^^33^

The usual nidus of infection for neck and chest areas is the teeth and tonsils.^34^^-^^42^ It is associated with high mortality. Minor trauma in our diabetic patient (C2) led to a host of response in the face and neck area. This type of presentation has not been reported in the literature. The involvement of perineum, thigh and abdominal wall similar to Fournier’s synergistic gangrene is common.^43^ In our patient, rectal examination was informative in establishing the cause of necrotizing fasciitis. It was the local manifestation of an end stage disease, i.e., carcinoma of rectum. Necrotizing fasciitis following C-section has been reported following the administration of NSAIDs in the immediate post-operative period.^44^^,^^45^

Periocular necrotising fasciitis is unusual due to the excellent blood supply of the facial region and only a few cases have been reported.^46^ In the early stages, it may be mistaken for cellulitis.^47^^,^^48^ A conservative debridement helped to retain the eyelid structures as much as possible.^49^^,^^50^ Though necrotizing fasciitis of the back of trunk and leg is not uncommon, association of chronic pancreatitis as in our case is unusual. Wounds could never be optimized and healing was poor, despite multiple debridements (in view of mal-absorption and malnutrition secondary to chronic pancreatitis), which were responsible for prolonged morbidity and mortality in our patient.

 Necrotizing fasciitis of scalp is rare, but can sometimes occur due to odontogenic infections.^21^^, ^^44^^, ^^51^^-^^59^ But in our patient, it followed trauma. The disease spread rapidly and on surgery, there was extensive necrosis of the pericranium and SMAS with induration of ear and neck; which took unusually long time to settle despite multiple debridements. Necrotising fasciitis of abdominal wall presenting with features of sub-acute intestinal obstruction as in (C7) has not been reported. The overall outcome was good in our patients as six out of seven survived, despite of septicemia and associated co-morbidities. The major hindrances to the effective treatment are accurate diagnosis and detection of underlying primary cause, if there are any. Though there is no antidote to arrest the chain of host response, which prolongs morbidity and risk of mortality, the disease can still be brought under control with aggressive surgical management and appropriate antibiotics.^60^^-^^62^

## CONCLUSION

Necrotising fasciitis is a life threatening infection. Often it begins following a minor etiology. The diagnosis is often delayed due to paucity of symptoms and cutaneous findings. It is often treated as a minor disease process. An early accurate diagnosis can be made with a high index of suspicion and bedside diagnostic confirmative tests, i.e. Andersen’s finger test. Sometimes involvement of unusual clinical sites and involvement of uncommon anatomical region may mislead the diagnosis in initial stages. In some patients, it may be the local manifestation of a systemic disease; which brings the hidden primary pathology to notice. In such situations, the treatment of primary cause is also mandated. The mortality and morbidity of the disease is high but if detected early and treated appropriately, good outcome can be expected.

## CONFLICT OF INTEREST

The author has no conflicts of interest to declare.
